# Description of Peripheral Blood Perfusion by Laser Speckle Contrast Analysis (LASCA) in ‘Early’ versus ‘Clinically Overt’ Systemic Sclerosis in Routine Clinics

**DOI:** 10.3390/diagnostics13091566

**Published:** 2023-04-27

**Authors:** Seppe Willems, Vanessa Smith, Steven Wallaert, Emanuele Gotelli, Tessa Du Four, Kaat Wyckstandt, Andrea Cere, Maurizio Cutolo

**Affiliations:** 1 Department of Rheumatology, Ghent University Hospital, 9000 Ghent, Belgium; sfriwill.willems@ugent.be (S.W.); tessa.dufour@ugent.be (T.D.F.); kaat.wyckstandt@uzgent.be (K.W.); 2 Department of Internal Medicine, Ghent University Hospital, 9000 Ghent, Belgium; 3 Unit for Molecular Immunology and Inflammation, Inflammation Research Center (IRC), Vlaams Instituut voor Biotechnologie (VIB), 9000 Ghent, Belgium; 4 Biostatistics Unit, Department of Public Health and Primary Care, Ghent University, 9000 Ghent, Belgium; steven.wallaert@ugent.be; 5 Laboratory of Experimental Rheumatology and Academic Division of Clinical Rheumatology, Department of Internal Medicine and Specialties, University of Genova, IRCCS San Martino Polyclinic Hospital, 16132 Genova, Italy; emanuele.gotelli@live.it (E.G.);

**Keywords:** systemic sclerosis, ‘early’ SSc, ‘clinically overt’ SSc, laser speckle contrast analysis (LASCA), Raynaud’s phenomenon

## Abstract

Objective: To investigate in an unselected, systemic sclerosis (SSc) cohort if baseline laser speckle contrast analysis (LASCA) peripheral blood perfusion (PBP) measurements differ between ‘early’ SSc (without skin involvement, or ‘limited’ SSc—LSSc) and ‘clinically overt’ SSc (with skin involvement, limited cutaneous SSc—LcSSc and diffuse cutaneous SSc—DcSSc) in routine setting. Methods: A group of twenty consecutive ‘early’ SSc patients and forty consecutive ‘clinically overt’ SSc patients (twenty LcSSc and twenty DcSSc) underwent clinical and LASCA examinations (to assess the peripheral blood perfusion [PBP] of both hands volar). Results: No statistically significant difference in adjusted PBP was found in the ‘early’ versus the ‘clinically overt’ group (*p* = 0.77) when adjusted for possible confounding factors (e.g., vasoactive medication, active smoking, history of DTL and disease duration). A wide variability was noted when observing the individual datapoints of each subset. Conclusion: This study with an unselected SSc population in daily routine, non-research setting, showed there was no difference in adjusted PBP at baseline between ‘early’ SSc and ‘clinically overt’ SSc when corrected for possible confounding factors. Interestingly a wide variation of individual datapoints were observed in each subset, which emphasizes the heterogeneity of SSc.

## 1. Introduction

Systemic sclerosis (SSc) is a rare multisystemic autoimmune connective tissue disease marked by microvascular damage and progressive fibrosis of the skin and internal organs [1,2]. Vascular involvement plays a crucial role in the pathogenesis of SSc, from the early stages of the disease to its late clinical complications such as digital ulcers (DUs) or critical ischemia. Furthermore, microvascular impairment represents the earliest morphological and functional hallmark of the disease, which is primarily clinically reflected in Raynaud’s Phenomenon (RP), which may precede the diagnosis of the disease by years [3,4,5,6,7]. According to LeRoy and Medsger criteria, ‘early’ SSc (without skin involvement; or ‘limited’ SSc—LSSc) is characterized by RP plus SSc-specific autoantibodies and/or a ‘scleroderma pattern’ on nailfold videocapillaroscopy (NVC), without any other distinctive characteristics of SSc [8]. SSc is considered ‘clinically overt’ (with skin involvement) when the disease flourishes with typical features such as fibrosis of the skin or internal organs and can be discerned into limited cutaneous SSc (LcSSc) and diffuse cutaneous SSc (DcSSc) [2,9].

Because of its significant role in SSc, the analysis of microvascular alterations is a crucial area of interest [10,11,12,13]. NVC is a valuable non-invasive and easily applicable technique for identifying and categorizing morphological peripheral microangiopathy, even in precursor stages of the disease (‘early’ SSc) [9,13]. It is regarded as a key tool in the diagnosis of SSc and has therefore been integrated in the 2013 American College of Rheumatology (ACR)/European League Against Rheumatism (EULAR) classification criteria [8]. Even though NVC is a reliable method for examining structural microvascular alterations, it is not feasible for examining the microcirculation functionally in daily settings in SSc [13]. Furthermore, NVC is postulated as a potential promising biomarker for future organ involvement in SSc [7,14].

Laser speckle contrast analysis (LASCA) has been proposed as a promising candidate to evaluate the peripheral blood perfusion (PBP) functionally and dynamically, over large skin areas, with particularly high spatial and temporal resolution [15,16,17,18]. It appeared to be a reliable method in the assessment of blood flow in SSc patients [17,18,19,20,21]. Of note, PBP values, measured by LASCA, were found to be significantly lower in SSc (LcSSc and DcSSc) patients compared to healthy subjects and in patients with previous or active DUs compared to those without [16,21]. Furthermore, PBP values showed a strong correlation with the progression of vascular damage evaluated by NVC [21]. Additionally, LASCA has been used to attest flow augmentation in trials with vasodilating medication [22,23,24].

In research setting (either following or during various forms of stress, such as the cold or occlusion test) differences in perfusion values between ‘early’ and ‘clinically overt’ SSc have been previously reported [25,26].

However, a potential role of LASCA in distinguishing between ‘early’ and ‘clinically overt’ SSc in daily routine, non-research practice has not been evaluated yet. Hence the aim of this pilot study was to investigate if LASCA can differentiate between the PBP of ‘early’ and ‘clinically overt’ SSc at baseline, in daily circumstances, in an unselected cohort of SSc patients.

## 2. Materials and Methods

### 2.1. Ethical Vote

The study protocol was approved by the Ethics Committee of the Ghent University Hospital (EC/2016/0175[BC15/1392]) and was conducted in accordance with the Declaration of Helsinki. All subjects gave their written informed consent for inclusion before they participated in the study.

### 2.2. Study Population

Sixty consecutive patients with the diagnosis of SSc who met the 2013 ACR/EULAR classification criteria for SSc and/or the 2001 LeRoy and Medsger classification criteria for LSSc, LcSSc and DcSSc were recruited [8,9]. ‘Early’ or LSSc was defined by the occurrence of RP, objectively documented by direct observation of clinical manifestations (any 2 of pallor, cyanosis or suffusion) or direct measurement of response to cold together with ‘scleroderma pattern’ at NVC or SSc-specific autoantibodies (anticentromere, anti-topoisomerase I—Scl-70, anti-fibrillarin, anti-PM-Scl, anti-fibrillin or anti-RNA polymerase I or III). LcSSc was defined by the criteria for LSSc in addition to skin thickness distal to elbows, knees and clavicles, while DcSSc was defined by the criteria for LSSc in addition to skin thickness proximal to elbows, knees and clavicles [8]. All patients visited the Ghent University Scleroderma Unit (GUSU) between September 2019 and December 2022 [8,9]. It should be noted that patients were permitted to continue taking their regular medications (including vasoactive therapy) as the study population was intended to resemble patients from day to day clinical practice as much as possible.

### 2.3. Data Collection

Data collection included demographic statement (e.g., age, gender), disease onset (defined by first non-RP sign/symptom), disease duration, presence of RP, autoantibody profile, modified Rodnan Skin Score (mRSS), current vasoactive therapy, status of smoking (active/past/never) and history of digital trophic lesions (DTL), including pitting scars and/or DU. The mRSS and the disease duration for ‘early’ SSc was set to zero because no distinctive characteristics of SSc, except the RP, were present.

### 2.4. Study Design

**Figure 1:**
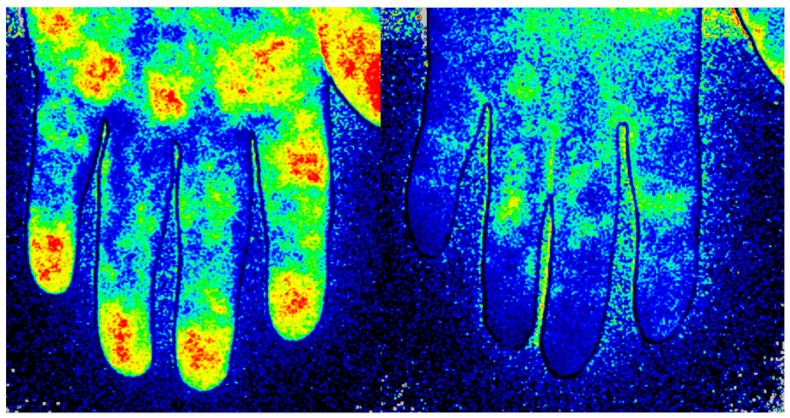
Imaging of the blood perfusion using laser speckle contrast analysis (LASCA) at the volar side of the hand in a healthy subject (**Left**) and in a patient with systemic sclerosis (**Right**).

**Figure 2:**
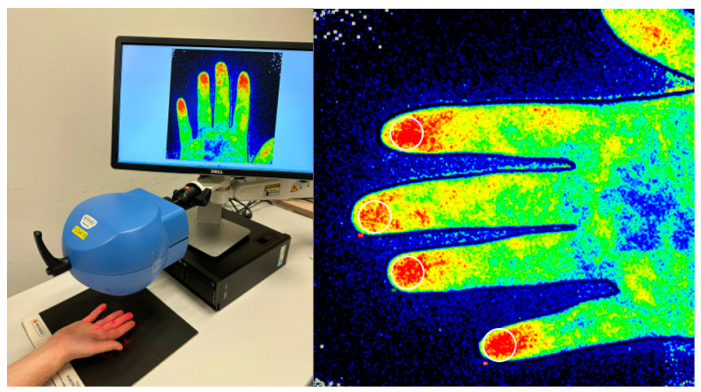
(**Left**) Measurement performance. (**Right**): An illustration of how to measure the mean peripheral distal blood flow. A ROI (circle area of 1 cm of diameter) is outlined at the 2nd–5th fingertip at the volar side.

LASCA recordings (Pericam PSI, Perimed, Jarfalla, Sweden) were conducted according to a pre-established standardized protocol [17,18]. All examinations were captured during a 30 s period, with a measuring area of 12 × 12 cm and a distance of 20 ± 0.5 cm from the scanner to the hand (Figure 1). Images were acquired under standardized instrumental and environmental conditions, after an acclimatization period of 20 min in which the participant remained in a calm, light- and temperature-controlled room [16,17,18]. Different regions of interest (ROIs) were created at the fingertips of each hand (ROI = circle area of 1 cm of diameter, placed at the 2nd–5th fingertip volar bilaterally), using the LASCA software (PIMSoft 15.1, Perimed AB) (Figure 2) [16]. The adjusted mean PBP of the ROIs was calculated and expressed in perfusion units [PU].

### 2.5. Statistical Analysis

To acquire the adjusted mean PBP, a linear mixed model was fit with a random intercept per patient together with a linear spatial correlation structure to capture the residual correlations between fingers. Fixed effect terms included subset, side hand (left/right), finger, vasoactive medication, active smoking, history of DTL and disease duration. Unpaired student’s *t*-tests were performed to compare a continuous variable between two groups. Categorical variables were compared via Chi^2^ test or Fisher’s exact test where appropriate. Significance level was set at 0.05 and no correction for multiple testing was applied. For descriptive purposes, absolute numbers with percentages were presented for categorical variables and means with standard deviation (SD) for continuous variables. Additional 95% confidence interval (CI) were given where needed. All data were analyzed using R, version 4.2.1 (R Core Team (2022) [27] and the nlme package (Pinheiro, J. et al. (2022) [28]).

## 3. Results

### 3.1. Study Population

A total of 60 SSc patients (15 men, 45 women; mean age 53 ± 12.6 years, mean disease duration 73.1 ± 89 months) were enrolled. The demographic and clinical data are shown in Table 1. Additionally the inclusion criteria of the ‘early’ SSc group, as defined by LeRoy (2001), can be found in Supplementary Table S1 [8]. In all patients RP was documented. A history of smoking was observed in 32 patients (53.3%) with 10 (16.6%) current smokers. SSc-specific antibodies were present in 29 patients (48.3%) with anti-topoisomerase-I as the most prevalent (14 patients, 23.3%). A total of 20 patients (33.3%) received vasoactive medication, of which 8 patients (40%) took multiple vasodilators. Among ‘clinically overt’ SSc a history of DTL was found in 16 patients (26.7%), 15 with pitting scars (25.0%) and 12 with DU (15.0%), respectively. When comparing baseline characteristics of the ‘early’ subset versus the ‘clinically overt’ subset the only significant difference was seen in the use of vasodilatory medication, which was found more frequently in the ‘clinically overt’ group. No other significant differences were observed (Table 2). Per definition, mRSS and history of DTL were not collected from ‘early’ SSc patients.

### 3.2. LASCA Examination

**Figure 3:**
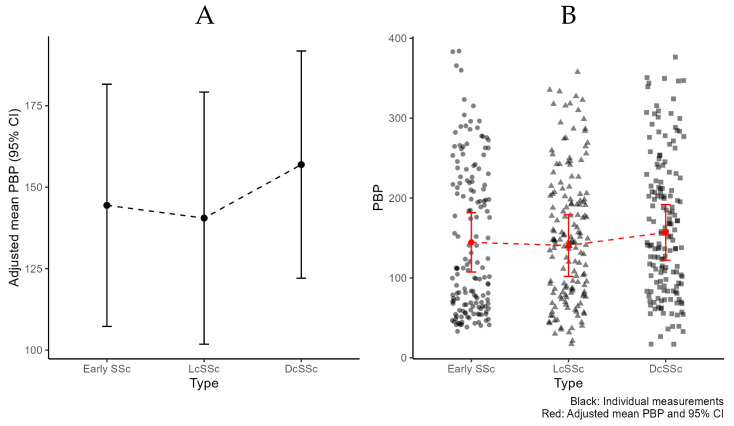
Peripheral blood perfusion (PBP) per subtype of systemic sclerosis, according to LeRoy and Medsger criteria 2001 [8]. (**A**): Adjusted mean PBP and 95% Confidence Interval (CI). (**B**): Additional individual measurements.

During statistical analysis visual inspection showed heteroscedasticity which was accounted for through weighted estimation. Details on the adjusted mean PBP for ‘early’ SSc versus ‘clinically overt’ SSc are provided in Table 3. When comparing the adjusted mean PBP at baseline in the ‘early’ versus the ‘clinically overt’ group, no statistically significant difference was found (144 vs. 150 PU, *p* = 0.77). Additionally, within the ‘clinically overt’ group no significant difference was noted between DcSSc and LcSSc (157 vs. 141, *p* = 0.53) (Figure 3A). A wide variability was observed between the individual measurements of each subgroup as seen in Figure 3B, with ‘early’ SSc ranging between 33 and 384 PU, LcSSc between 17 and 358 PU and DcSSc between 17 and 376 PU.

## 4. Discussion

To our knowledge, this pilot study describes for the first time the PBP, measured by LASCA, in ‘early’ (LSSc) versus ‘clinically overt’ SSc (LcSSc and DcSSc) in a cohort of un-selected consecutive SSc patients in daily routine, non-research setting. Corrections were made to minimize potential confounding factors, in particular for vasoactive therapy (calciumchannel blockers, phosphodiesterase 5 inhibitors and prostaglandin E2—alprostadil in our cohort), and to enable a more accurate interpretation of the obtained PBP measurements. After accounting for these adjustments, it can be concluded that in our monocentric cohort the mean PBP did not significantly differ between ‘early’ SSc and ‘clinically overt’ SSc, suggesting impaired flow, already in precursor stages of SSc. This is corroborated by the fact that in a research setting differences in perfusion values between ‘early’ and ‘clinically overt’ SSc have had been previously reported [25,26]. Notably, the perfusion in research setting is being evaluated in laborious circumstances (either following or during various forms of stress, such as cold or occlusion test), not facilitating its use in routine clinics [25,26].

Another reason may be the large proportion of vasodilation having been taken by our DcSSc patients. This may have artificially augmented the mean flow in the DcSSc group weaning out any significant differences in flow in between groups. This option is backed up by the fact that in studies where no vasodilation is used the flow in DcSSc is lower than in LcSSc [19,21,29,30].

Interestingly, a wide variation of individual measurements was seen in each subgroup (Figure 3B), which emphasizes the heterogeneity of SSc. Future studies will have to elucidate whether baseline LASCA may be of use as an intra-subject follow up assessment of flow, in the same patient over time.

Although we were unable to confirm the hypothesis of our study, this does not mean the end for further research on the applicability of LASCA in everyday settings. It is important to stress that the possible added value of this tool as suggested by previous research cannot be disregarded [16,17,18,19,20,21,22,23,24,29,30,31].

## 5. Conclusions

In conclusion, our pilot study with a day-to-day SSc population was unable to discriminate between ‘early’ and ‘clinically overt’ SSc. Larger cohorts of patients are needed to confirm or deny these preliminary observations.

Moreover, future investigations are needed to study the intra-individual changes over time of PBP in SSc patients and if they correlate with peripheral vascular clinical manifestations.

## Figures and Tables

**Table 1:** Baseline characteristics of the study population (*n* = 60).

Baseline Characteristics General				
Age (Years), Mean ± SD			53 ± 12.6	
Gender (♂/♀), *n* (%)			15 (25)/45 (75)	
Disease duration (months), mean ± SD			73.1 ± 89	
Raynaud’s phenomenon, *n* (%)			60 (100)	
Smoking, *n* (%)			32 (53.3)	
Past smoking, *n* (%)			22 (36.6)	
Active smoking, *n* (%)			10 (16.6)	
mRSS, mean ± SD			12 (9.7)	
LeRoy subset, ‘Early’ SSc/LcSSc/DcSSc, n (%)			20 (33.3)/20 (33.3)/20 (33.3)	
**Baseline characteristics per subset**				
Subset (*n*)	Total (*n* = 60)	‘Early’ SSc (*n* = 20)	LcSSc (*n* = 20)	DcSSc (*n* = 20)
SSc-specific Ab, *n* (%)	29 (48.3)	7 (35.0)	11 (55.0)	11 (55.0)
NVC scleroderma pattern, *n* (%)	54 ^a^ (90)	17 (85)	20 (100)	17 ^a^ (85)
Anticentromere Ab, *n* (%)	13 (21.7)	4 (20.0)	8 (40.0)	1 (5.0)
Anti-topoisomerase-I Ab, *n* (%)	14 (23.3)	2 (10.0)	3 (15.0)	9 (45.0)
Anti-RNA-polymerase III Ab, *n* (%)	2 (3.3)	1 (5.0)	0 (0.0)	1 (5.0)
Vasoactive medication, *n* (%)	20 (33.3)	3 (15.0)	6 (30.0)	11 (55.0)
CCB, *n* (%)	11 (18.3)	3 (15.0)	4 (20.0)	4 (20.0)
PDE5-i, *n* (%)	1 (1.7)	0.0 (0.0)	0.0 (0.0)	1 (5.0)
CCB + PDE5-i, *n* (%)	3 (5.0)	0.0 (0.0)	0.0 (0.0)	3 (15.0)
CCB + PGE1, *n* (%)	2 (3.3)	0.0 (0.0)	1 (5.0)	1 (5.0)
PDE5-i + PGE1, *n* (%)	2 (3.3)	0.0 (0.0)	1 (5.0)	1 (5.0)
CCB + PDE5-i + PGE1, *n* (%)	1 (1.7)	0.0 (0.0)	0.0 (0.0)	1 (5.0)
History of DTL, *n* (%)	16 (26.7)	0.0 (0.0)	5 (0.25)	11 (55.0)
History of pitting scars, *n* (%)	15 (25.0)	0.0 (0.0)	4 (20.0)	11 (55.0)
History of DU, *n* (%)	12 (20.0)	0.0 (0.0)	3 (15.0)	9 (45.0)

^a^ 3 missing values. Ab: antibody; CCB: calcium channel blocker; DcSSc: diffuse cutaneous systemic sclerosis; DTL: digital trophic lesion; DU: digital ulcer; Early: ‘early’ systemic sclerosis (limited systemic sclerosis); LcSSc: limited cutaneous systemic sclerosis; mRSS: modified Rodnan skin score; NVC: nailfold videocapillaroscopy; PDE5-i: Phosphodiesterase-5-inhibitor; PGE1: prostaglandin E1; SSc: systemic sclerosis.

**Table 2:** Comparison of baseline characteristics for ‘early’ SSc versus ‘clinically overt’ SSc.

Characteristics	‘Early’ SSc (*n* = 20)	‘Clinically Overt’ SSc (*n* = 40)	*p*
Females, *n* (%)	17 (85.0)	28 (70.0)	0.34
Age (years), mean (SD)	48.8 (13.9)	55.1 (11.5)	0.07
Active smoking	1 (5.0%)	9 (22.5%)	0.14 ^a^
SSc-specific Ab, *n* (%)	7 (35.0)	22 (55.0)	0.24
mRSS, mean (SD)	0 (0.0)	12 (9.7)	
Vasoactive medication, *n* (%)	3 (15.3)	17 (42.5)	0.04 ^a^
History of DTL, *n* (%)	0 (0.0)	16 (40.0)	

^a^ Fisher’s exact test. Ab: antibody; DTL: digital trophic lesion; Early: ‘early’ systemic sclerosis (limited systemic sclerosis); mRSS: modified Rodnan skin score; SSc: systemic sclerosis.

**Table 3:** Adjusted mean peripheral blood perfusion comparison.

					Statistical Significance			
	‘Early’ (*n* = 20)	‘Overt’ (*n* = 40)	LcSSc (*n* = 20)	DcSSc (*n* = 20)	‘Early’ vs. ‘Overt’	‘Early’ vs. LcSSc	‘Early’ vs. DcSSc	LcSSc vs. DcSSc
PU, mean	144	150	141	157	*p* = 0.77	*p* = 0.89	*p* = 0.62	*p* = 0.53
95% CI	107–182	124–175	102–179	122–192				

DcSSc: diffuse cutaneous systemic sclerosis; Early: ‘early’ systemic sclerosis (limited systemic sclerosis); LcSSc: limited cutaneous systemic sclerosis; Overt: ‘clinically overt’ systemic sclerosis discerned into LcSSc and DcSSc; PU: perfusion unit; SSc: systemic sclerosis.

## Data Availability

The data presented in this study are available on request from the corresponding author.

## Supplementary Materials

The following supporting information can be downloaded at: https://www.mdpi.com/article/10.3390/diagnostics13091566/s1, Table S1. Baseline characteristics of the ‘early’ SSc group (*n* = 20).
