# Supplementary material for: Description of Peripheral Blood Perfusion by Laser Speckle Contrast Analysis (LASCA) in ‘Early’ versus ‘Clinically Overt’ Systemic Sclerosis in Routine Clinics
Source: Diagnostics (Basel). 2023 Apr 27;13(9):1566. doi: 10.3390/diagnostics13091566 (PMC10177938; doi:10.3390/diagnostics13091566)
Supplement: Supplementary file 1 [file diagnostics-13-01566-s001.zip › diagnostics-2375436-supplementary.pdf]

Supplementary File

Table S1. Baseline characteristics of the ‘early’ SSc group (n = 20).

**Baseline characteristics of the ‘early’ SSc group**	
Raynaud’s phenomenon, n (%)	20 (100)
Puffy fingers, n (%)	7 (35.0)
NVC scleroderma pattern, n (%)	17 (85.0)
SSc-specific Ab, n (%)	7 (35.0)
Anticentromere Ab, n (%)	4 (20.0)
Anti-topoisomerase-1 Ab, n (%)	2 (10.0)
Anti-RNA-polymerase III Ab, n (%)	1 (5.0)
**Inclusion criteria ‘early’ SSc as defined by LeRoy (2001) (8)**	
No scleroderma pattern + SSc specific Ab, n (%)	3 (15.0)
Scleroderma pattern + no SSc specific Ab, n (%)	13 (65.0)
Scleroderma pattern + SSc specific Ab, n (%)	4 (20.0)
Total, n (%)	20 (100)

Ab: antibody; Early: ‘early’ systemic sclerosis (limited systemic sclerosis); NVC: nailfold videocapillaroscopy; SSc: systemic sclerosis.
